# Case of Epilepsy, Induced by a Blow upon the Head, Successfully Treated

**Published:** 1843-02-04

**Authors:** Isaac Parrish


### CASE OF EPILEPSY,

### INDUCED BY A BLOW UPON THE HEAD, SUCCESSFULLY TREATED.

BY ISAAC PARRISH, M. D.

[*Read before the Philadelphia Pathological Society.*]

H. T———, the subject of the present note, is a young man of about 20 years of age, tall and of slim stature, and of strumous constitution, having been at one time affected with necrosis of the tibia, from which he had recovered, with the loss of several portions of bone.

While engaged in his occupation, as clerk of a store, he had occasion to get upon a high counter, and in suddenly rising upon his feet, from a stooping position, struck his head with violence against the end of a gas-pipe, which projected from the ceiling. The blow was received upon the top of the head, two or three inches to the left of the sagittal suture.

He fell back in a state of insensibility, and, in a few moments, passed into a convulsion. A physician was immediately sent for, but when he arrived consciousness was returning; and nothing was prescribed in the way of medicine; the patient was cautioned, however, against undue exertion, &c. After the fit, severe headache continued, but the patient considered himself well enough to walk home, a distance of several squares.

On reaching the door of his chamber, in the third story, he uttered a loud scream, and fell prostrate on the floor in a violent convulsion. I was with him in a few minutes, while the attack was still upon him, and have seldom witnessed a more severe fit. The face was deeply flushed, head hot, eyes injected, pulse tense, with frothing at the mouth, and general muscular disturbance.

Iced water was freely poured over the head, and sinapisms were applied to the feet, and in a few minutes the attack subsided.

A large number of leeches were now applied to the temples, and the sinapisms were continued to different parts of the extremities, until the stupor following the convulsion had entirely passed away.

As consciousness returned, the pain in the head was still the subject of complaint; this, however, was greatly relieved by the leeching; the patient became calm and rational, and detailed with precision the particulars of the accident.

In about an hour, however, he broke out into incoherent conversation, screaming and laughing by turns, with hot head and flushed face; this quickly passed over under the use of cold applications, and he sank into a quiet, natural sleep. During this visit, the point of injury was carefully examined by shaving the head over the part, but nothing but a slight contusion could be discovered—the bone not appearing to be injured.

The accident occurred Seventh mo. (July) 31st, 1839, in the afternoon. During the night the patient slept calmly, but on the following day he had several attacks of severe headache, coming on suddenly, and attended with screaming, laughing, &c., as above described. During several of these, he was insensible for a few minutes; but was generally easy and tranquil in the intervals between them.

He took several doses of a decoction of senna and Sulph. Magnes. during the day, which purged him freely; the cold applications were continued to the head, and he was kept upon a low diet.

On the following day, Eighth mo. (August) 2d, the attacks were less frequent and severe, and in two or three days they subsided altogether. The patient was carefully excluded from all causes of excitement, being kept quiet in his chamber, and restricted in diet, until all headache and uneasiness had entirely left him, when he was allowed to go abroad cautiously, without, however, engaging in any business, and being restrained as far as possible from every thing which might excite his nervous system.

For a period of eight months the patient escaped a renewal of the disease; and it was hoped that he had entirely recovered from the predisposition. He suffered, however, from another attack in the Fourth mo., (April,) 1840. It came on during the exercise of singing at a place of worship; and, from what I could learn, resembled very much those which had preceded it. From this time the susceptibility to the disease increased, and the attacks recurred at shorter intervals. Any sudden fright was apt to induce it; on several occasions the noise of the firemen and the ringing of the bells brought on a paroxysm. The patient was sent to the country with a view of improving his general health, and of separating him from those causes which seemed to excite his malady.

He remained out of town several weeks, during which time he was clear of the convulsions; on his return, however, they recurred, and were so frequent and distressing as to cause his friends great anxiety, and to render it unsafe for him to go abroad alone.

It was now remarked, by those who witnessed these attacks, that they were immediately preceded by a shoot of pain in that portion of the head which had been the seat of injury; the patient would seize his hair over this spot, and pull it forcibly, as if in agony, and then pass into a convulsion.

No pain in the head was complained of at any other time, nor did the patient seem aware that any connection existed between these attacks and the blow he had received. On close examination of the injured part, however, it was very evident that there was a sensitive point, although nothing externally indicated it. Pressure upon a spot about the size of a quarter of a dollar piece, a little to the left of the sagittal suture, caused severe pain and general nervous agitation, and indicated with sufficient clearness, the close connection between the convulsions and the injury which this part had sustained. This was evidently the point from which the *aura* arose, and at once assumed an important place in the subsequent treatment of the case.

A consultation was now requested, and that excellent and experienced surgeon, Dr. Thomas T. Hewson, joined me. After a careful review of the history of the case, it was determined to address our remedies chiefly to the original seat of injury; it was believed that the acute pain on pressure, and the evident suffering prior to a paroxysm, felt at this spot, furnished sufficient indications for such a course.

Eighth mo. (Aug.) 1st, 1840—just one year after the reception of the injury—assisted by Dr. Hewson, I made an incision, about two inches long, directly through the tender portion of the scalp, and down to the bone. Several issue peas were inserted between the edges of the wound, and secured in their places by plaster. In a few days purulent discharge took place, and a constant drain was thereby established from this irritated surface. A course of constitutional treatment, with tonics, salt bath, &c., was at the same time instituted, and we had the gratification of witnessing a rapid amendment in our patient; no return of pain or convulsions occurred, and his general health improved.

About a month after the incision into the scalp, the patient was attacked with severe periostitis in the limb which had previously been the subject of necrosis. Leeching, anodyne plasters, &c., relieved this affection in a degree.

At the end of seven weeks, the soreness in the scalp having entirely disappeared, it was deemed safe to remove the peas, and heal up the issue. A seton was passed over the tibia, which continued painful at times; this was kept discharging for several months, until all pain in this part had disappeared.

Our attendance upon the patient now ceased, although we did not consider him safe from a recurrence of the convulsions. He has, however, escaped entirely up to this time—a period of between two and three years—thus giving us the opportunity of reporting his case as cured. When we consider the frequency of the attacks prior to the issue in the scalp, and the entire immunity from them since that time, notwithstanding the constant exposure of the patient to those causes which formerly induced them, we must regard this procedure as a highly interesting and important curative measure—at least in the present instance.

*Remarks.*—The great obscurity in which the pathology of Epilepsy is involved, and the inefficiency of all known medical means in its treatment, at least in the large proportion of cases, renders the report of every case, in which remedies have proved availing, a matter of some importance.

The case now presented offers an interesting example of that form of Epilepsy termed by the old writers, *sympathetic*, in which the brain appears to be involved secondarily—the paroxysm taking its rise from a point *without* the cranium. This variety has always been considered more manageable than that which has its origin in some hidden cause *within* the brain, whether induced by hereditary predisposition, or from a constitutional vice of any kind. Hence Hippocrates has sagaciously remarked that those epileptics are very hard to be cured "in whom the disease affords no sign from what part of the body it takes its origin;" and Celsus has said with equal propriety—"that if the whole body is affected at once, and no sense of the approaching fit is felt in any particular part, but the person falls down unexpectedly, of whatever age he is, he can hardly be cured." Many similar expressions might be quoted from ancient authors, tending to show the importance which they attached to this distinction between diopathic and sympathetic epilepsy—a distinction which appears to have been too much overlooked by modern practitioners, except in cases where it is so obvious as to force itself upon our attention, as in fits occurring in infants from dentition, from indigestible matters in the alimentary canal, &c. Points of local irritation upon the surface of the body may be as influential in inducing an epileptic paroxysm, as internal causes, and, by removing them, we may be equally successful in putting a period to the disease.

Hence the importance of close inquiry into the history of each case, and if a centre or point be discovered, from which the aura epileptica takes its origin, our remedies may be directed to it with a hope of success. The application of blisters, issues, and even of the cautery to such diseased parts, are strongly recommended by the old authors, and many cases are reported in which they have been successful.

In some instances, the occurrence of a spontaneous discharge of matter from the head, the appearance of an eruption, or an accidental wound of the scalp, have accomplished a cure. Thus, we find in Van Swieten's Commentaries the case of a French nobleman related, who, being troubled with an epilepsy, took a journey into Italy, in order to consult the most skilful physicians there; but being plundered by robbers upon the road, and very much wounded, he was left for dead: besides other wounds, he had received a very large one in his forehead, which carried off a great part of the bone. After a long time he was cured of this wound; and at the same time was freed of the epilepsy, which used to return upon him every month."*

The use of the trepan in certain cases of epilepsy, following injuries of the head, was also recommended by the ancients—especially when the symptoms indicated disease of the bone, or the extravasation of humours under the skull, or pressure on the brain, from a depressed fragment. This operation has been revived in this country by Dr. Dudley, of Lexington, who has reported several very interesting cases of cure after its performance. Dr. D. very judiciously restricts the resort to so serious a measure, to those cases only in which there is an evident depression of bone, or a morbid condition of it, produced by violence or other causes, leaving, thereby, but little doubt as to the point to which the instrument should be applied, and of the practicability of removing the cause upon which the cerebral excitement depends.

Dr. Dudley also believes that chronic inflammation of the pericranium is not an unfrequent result of contused wounds of the scalp, which have been healed too rapidly by the first intention; and that many unpleasant and alarming symptoms are the consequence of this practice.*

The case now narrated would seem to favour the idea, that chronic inflammation of the pericranium may even induce epilepsy, and that the establishment of a drain over the diseased surface affords the best means of relief.

Nor is it irrational to suppose, that the same practice might answer even in cases where the bone itself is diseased, without a resort to the trephine, provided the internal table were not involved in the mischief, and no pressure existed upon the brain.

Painful affections of particular nerves, coming on in paroxysms, and arising from disease in the trunk of the nerve, whether induced by injury or from other causes, are sometimes the cause of convulsions, which, if not arrested, may become habitual. Under these circumstances, division of the diseased trunk is obviously indicated, and has been long practised. A very curious case of this kind is related in the learned Commentaries just quoted: "A woman, of thirty-eight years of age, had been twelve years subject to the epilepsy: in the beginning of the disease she had a paroxysm every month; afterwards it so increased, that she suffered four or five strong fits every day, each of which lasted for an hour and upwards; whence, being rendered quite dull and stupid, she was no longer able to take care of her family. All kinds of remedies were used without the least success, the disease still growing worse.

In the mean time, the paroxysm always began in the leg, about the lower part of the gastrocnemii muscles; immediately it flew up to her head; and then she fell down violently convulsed, and foaming at the mouth.

A physician, who was present during the time of paroxysm, compared the leg affected with the other, and he could not distinguish any difference between them: however, he boldly thrust in a scalpel, to the depth of about two inches, and in the bottom of the wound he found a hard, cartilaginous body, somewhat larger than a pea; he separated it from the muscles, and found that it rested upon a nerve: cutting the nerve, he laid hold of that heterogeneous body, and he pulled it out; this was no sooner done, but immediately the patient recovered out of the fit, saying that she was very well, and afterwards lived quite free of this terrible disease, and recovered her former vigour both of mind and body."†

* Van Swieten's Commentaries, vol. x, p. 425.

* Transylvania Journal of Medicine, vol. i.

† Van Swieten's Commentaries, vol. x., p. 438.

The facts thus hastily thrown together, sufficiently prove the position, that a small point of local irritation, which might readily escape notice, may prove the source of this terrible disease, and that it becomes the physician to investigate closely the history of each case.

That many cases of idiopathic epilepsy occur, which are entirely beyond all known means of cure; is lamentably true; while it is possible that cases deemed hopeless might be relieved, were physicians more accurate in scrutinizing them.

It may be remarked, in addition, that the practice of making an incision through the scalp over the sagittal suture, and of inserting the issue peas, with a view of establishing steady counter-irritation in this situation, has been employed for some years past by Dr. Charles Evans in the treatment of chronic affections of the brain, with very satisfactory results. The extensive opportunities enjoyed by Dr. Evans for observation on these diseases, as physician of a large insane hospital, renders his experience upon the question very valuable.

